# Accuracy of acetabular cup positioning in robotic-assisted total hip arthroplasty: a CT-based evaluation

**DOI:** 10.1051/sicotj/2024057

**Published:** 2024-12-20

**Authors:** Ashish Singh, Purushotam Kumar, Kanukuntla Kalyan, Akash Chandrashekar Gundalli, Sudhir Shankar Mane, Himanshu Swarnkar, Lavanya Singh

**Affiliations:** 1 Anup Institute of Orthopaedics & Rehabilitation G75-77, P.C. Colony Kankarbagh, Patna Bihar 800020 India; 2 The Hazeley Academy Emperor Drive, Hazeley Milton Keynes MK8 0PT United Kingdom

**Keywords:** Total hip arthroplasty, Acetabular cup positioning, Robotic-assisted surgery, Preoperative planning, Intraoperative planning

## Abstract

*Introduction*: Robot-assisted instrumentation during total hip arthroplasty (THA) has the potential to improve acetabular cup positioning. This study aimed to evaluate the precision of robotic-arm-assisted THA (rTHA) and assess whether the system can provide accurate cup positioning comparable to conventional THA (cTHA). *Methods*: A single-center prospective cohort study consisting of 151 patients who underwent THA (108 rTHA and 43 cTHA). The robotically assisted system was used to match the postoperative computed tomography (CT) image of the pelvis with the planned and intraoperative anatomical landmarks. The cTHA cohort underwent hip replacement using the standard manual procedure, with acetabular component locations assessed during and after surgery. *Results*: The rTHA cohort was significantly younger, but no other significant differences were found between the two cohorts in preoperative baseline data. In rTHA cohort, the planned inclination (40.0 ± 0.3°) closely matched the intraoperative (40.2 ± 2.7°; *p* = 0.54) and postoperative (40.7 ± 4.0°; *p* = 0.07) measurements. However, anteversion showed a significant increase from planned (19.4 ± 1.5°) to postoperative CT scan (28.7 ± 7.0°; *p* < 0.001). There was evidence of proportional bias in the measurements (*p* < 0.001). In the cTHA cohort, the mean inclination (43.1 ± 5.1°) did not show any significant change between the preoperative plans and postoperative assessments (*p* = 0.12); however, there was a remarkable change in the mean anteversion (17.6 ± 6.4°) between postoperative measurements and the preoperative plans (*p* < 0.001). The average anteversion in the preoperative plans did not differ remarkably between the rTHA and cTHA cohorts. However, the average inclination was substantially different between the two cohorts (*p* < 0.001). Both groups had no significant differences in the proportion of cups outside the referenced safe zones. *Conclusion*: The results suggest that while robotic-assisted guidance ensures consistent cup inclination, there may be more variability in achieving the planned anteversion, which warrants further investigation into the factors influencing postoperative changes in acetabular orientation.

## Introduction

Total hip arthroplasty (THA) is a commonly performed procedure for patients with osteoarthritis of the hip joint. Proper positioning of the femoral and acetabular components is vital for optimal outcomes. Despite various technical advances, the placement of the acetabular component in primary THA is still a challenging issue.

The acetabular cup must have the ideal combined anteversion and inclination to distribute joint dynamics evenly throughout the articular surfaces. Errors might cause occasional leg length disparity [1], instability [2], dislocation [3], excessive wear, and osteolysis, [4, 5]. Non-concentric loading patterns in metal-on-metal bearing surfaces may cause metallosis. Additionally, cup malpositioning can lead to squeaking, edge wear, and severe soft tissue destruction [6, 7].

The most prominent study on this subject published in 1978 by Lewinnek et al., recommended a “safe range” of 40° of inclination (±10°) and 15° of anteversion (±10°) [8]. In a recent study, Callanan et al. proposed that the evaluation of acetabular component placement after THA should employ a modified, restricted safe zone. 5° to 25° of anteversion and 30° to 45° of inclination were the parameters used to establish this new zone [9]. Many factors, including obesity, surgical approach, surgeon volume, and deformity of the pelvis or proximal femur make manual intraoperative evaluation of cup positioning challenging [4, 8, 9]. Newer technologies and techniques have been developed as a result of these drawbacks with the conventional THA (cTHA) approach of free-hand acetabular component placement. The orientation of the acetabular cup can be validated using C-arm fluoroscopic imaging, computer-assisted navigation, and landmarks such as the transverse acetabular ligament (known as the Beverland technique) [10–12].

Novel technologies have been created to enhance the precision of cup positioning and reduce these limitations of cTHA. Several articles have demonstrated that robot-assisted THA can enable surgeons to enhance the accuracy of cup positioning compared with cTHA [13–15]. One such technology is robotic-assisted THA, which creates a patient-specific 3D model of the proximal femur and pelvis using a preoperative computed tomography (CT) scan. Landmarks are registered and mapped during the procedure so that the software can pinpoint the patient’s precise location in space. To accomplish the best possible cup placement in terms of medialization, anteversion, and inclination, the robotic console directs the surgeon during reaming and cup positioning. The desired position of the cup is decided by the operating surgeon and can be manipulated intraoperatively as the inclination and version of the acetabulum are patient-specific. In adopting the switch from conventional to robot-assisted THA, surgeons have some queries in mind. Some of these concerns are whether the rTHA approach achieves the targeted version and inclination as planned as well as the cost implication of the robotic system.

This study aimed to evaluate the reliability and precision of the MAKO robotic system during THA. The main research question was: Do the values of cup version and inclination measured in the postoperative CT scans correlate with the intraoperatively measured and preoperatively planned values? Second, we compared the accuracy of cup placement between rTHA and cTHA done by the same surgeon and analyzed the differences.

## Materials and methods

### Study design

This single-center prospective cohort study started after receiving Institutional Review Board and ethical committee approval. A written informed consent was obtained from all patients. Patients who underwent THA between August 2020 and February 2024 in this center and who gave informed consent were included in this study. A single fellowship-trained arthroplasty surgeon who performs at least 200 THA operations annually and has substantial expertise with the RIO^®^ (Robotic Arm Interactive Orthopaedic System, MAKO Surgical Corp., Davie, FL) operated on all the patients. The work has been documented in compliance with the STROCSS guidelines [16].

### Inclusion and exclusion criteria

Patients who underwent primary cementless THA, gave consent to participate in the study, and agreed to undergo a postoperative CT scan were included in the study. Patients undergoing revision THA and cemented THA were excluded from the study.

### Preoperative preparation

Preoperative radiographic assessments including CT scans of bilateral knees and hips were imported into the MAKO system to generate a three-dimensional template. The acetabular cup can be repositioned and adjusted by the surgeon in several systemic planes. In the preoperative plan, all acetabular cups were intended to be positioned in 20° anteversion and 40° inclination. The express interface of the MAKO software was used for this process. Based on the intraoperative assessments, the surgeon minimally modified the cup placement in certain individuals to optimize stability and range of motion. In cTHA, we used an inclinometer to measure cup inclination. Following Beverland’s technique [12], we used the transverse acetabular ligament as a landmark for assessing anteversion, while also aiming for optimal coverage of the cup.

### Surgical procedure

The procedures were carried out by a single surgeon using the posterolateral approach with the patient in a lateral decubitus position. The surgeon attached the pelvic arrays and then proceeded with the skin incision and preparatory exposure. The distal and proximal femoral checkpoints were recorded to determine the hip offset and preoperative leg length before hip dislocation. The surgeon then performed the femoral neck osteotomy after dislocating the joint. The superior, anterior, and posterior rims of the acetabulum were shown to include three markers. Acetabular components used were either the Trident^®^ PSL^®^ Trident^®^ Hemispherical shell, or the Tritanium™ shell (Stryker, Fort Lauderdale, FL). These implants had a porous ingrowth surface and were underpinned by a concentric metal structure. In each case, a BIOLOX^®^ delta ceramic head or a cobalt-chromium femoral head was utilized in opposition to polyethylene. The intended acetabular cup position depending on the optimal coverage (targeted for 40° inclination and 20° anteversion) was gained from each plan preoperatively. The preoperative planning software’s assessment of native anteversion served as the basis for determining the acetabular version. This was further adjusted based on clinical factors such as patient laxity, sex, and specific functional needs, alongside the goal of achieving optimal cup coverage. Acetabular preparation was done first followed by femoral preparation and broaching. The RIO^®^ robotic-arm-assisted system recorded each patient’s raw electronic session file during the surgical operation. The following intraoperative information was taken from each session file: laterality, the final intraoperative positioning, and verification (anteversion and abduction of the acetabular cup), which was completed using the probe and recorded.

### Postoperative radiographic measurements

**Figure 1:**
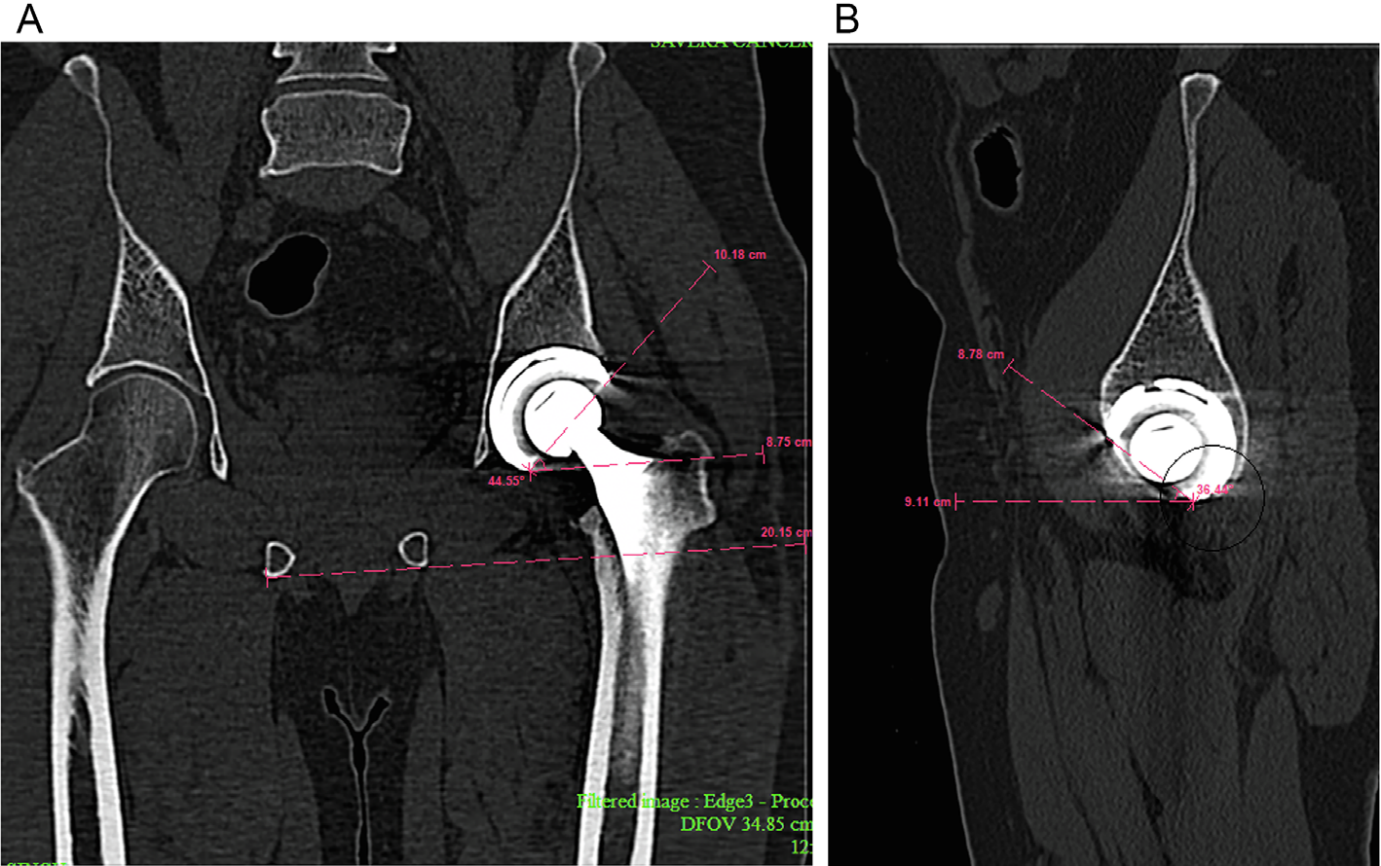
(A) Meyer’s method in the estimation of the inclination of the acetabular cup. (B) Meyer’s method in the estimation of the anteversion of the acetabular cup.

A postoperative CT scan was performed for each patient on the postoperative day six (±2 days) to validate the accurate positioning of the cup. The inclination and anteversion angles were measured using Meyer’s method [17]. This is illustrated in Figure 1. Two methods were used to assess the overall accuracy of acetabular cup implantation. One method produced data in scatter plots resembling those of Callanan et al. [9] and estimated the incidence of acetabular cups placed within a specified safe zone for both anteversion and inclination. Using the information from each component’s postoperative CT scan and the discrepancy between the expected and actual positions, the second method computed a 95% confidence interval. This method can only be applied in situations where each component’s precise intended placement is known, as with rTHA. A confidence interval computed in this way is called a predictive interval, as explained by Altman et al. [18], and can be utilized to forecast future performance when utilizing a specific technique or tool. Regarding the agreement between the postoperative CT scans radiographic analysis and preoperative plan, as well as between the intraoperative robotic-arm data and preoperative plan, a predictive interval was determined.

### Definitions

The angle formed by the cup’s long axis and the line joining the two teardrops on either side was defined as the inclination of the cup [2]. The anteversion of the cup was defined as the angle between the short and long axes of the ellipse projected by the cup. Plotting each patient’s anteversion and inclination allowed comparison with the Lewinnek et al. (anteversion, 5–25°; inclination, 30–50°) and Callanan et al. (anteversion, 5–25°; inclination, 30–45°) safe zones [8, 9].

### Data analyses

Data analysis was performed using IBM SPSS Statistics for Windows, Version 25.0 (IBM Corp Armonk, NY). Categorical data were reported as counts and percentages while continuous data were expressed as mean ± SD. Categorical cohorts were compared using the chi-square (*χ*^2^) test, while continuous variables were compared using the *t*-test. Paired *t*-tests were used to assess differences in preoperative, intraoperative, and postoperative measurements. Scatterplots and a 95% predictive interval were used to assess the position and precision of the acetabular cups as described. For all cases, a *p*-value < 0.05 was considered significant.

## Results

### Characteristics of the patients

A total of 151 patients were involved in this study; 108 patients in the rTHA cohort and 43 patients in the cTHA cohort. Table 1 provides the socio-demographic and clinical profile of the patients. The mean (±SD) age of individuals in the rTHA cohort was 44.65 (±14.27) years compared to 53.88 (±13.67) years among patients in the cTHA cohort (*t* = −3.70; *p* < 0.001). There were no significant differences in the preoperative baseline data of the individuals in the two cohorts according to their sex, BMI, surgical side and indication for the THA as shown in Table 1.

**Table 1:** Socio-demographic and clinical profile of the study participants.

Variables	rTHA	cTHA	*χ*^2^/*t*	*p*-value
	*n* (%)	*n* (%)		
Total	108	43		
**Age group**			10.4	0.006
<40	39 (36.1)	8 (18.6)		
41–65	63 (58.3)	26 (80.5)		
>65	6 (5.6)	9 (20.9)		
Mean ± SD	44.65 ± 14.27	53.88 ± 13.67	−3.7	<0.001*
**Sex**			0.0	0.98
Female	40 (37.0)	16 (37.2)		
Male	68 (63.0)	27 (62.8)		
**BMI (kg/m** ^**2**^ **)**				
≤24.9	64 (59.3)	26 (60.5)	0.02	0.89
25>	44 (40.7)	17 (39.5)		
Mean ± SD	24.04 ± 3.66	24.41 ± 1.66	−0.63	0.53*
**Surgical side**			0.01	0.97
Left	53 (49.1)	21 (48.8)		
Right	55 (50.9)	22 (51.2)		
**Diagnosis**			9.75	0.08
Avascular necrosis	28 (25.9)	13 (30.2)		
Osteoarthritis	25 (23.1)	7 (16.3)		
Ankylosing spondylitis	8 (7.4)	3 (7.0)		
Rheumatoid arthritis	13 (12.0)	8 (18.6)		
Dysplasia	22 (20.4)	2 (4.7)		
Others	12 (11.1)	10 (23.3)		

SD = Standard deviation; rTHA = robotic-assisted total hip arthroplasty; cTHA = conventional total hip arthroplasty; * = *p*-values based on *t*-test.

### Preoperative, intraoperative, and postoperative assessment of the cup angles and positions

Table 2 summarizes the mean angles of the acetabular cup anteversion and inclination recorded in the preoperative plans, postoperative assessments, and intraoperative robot-assisted records. In the rTHA cohort, the mean anteversion and inclination angles in the preoperative plan were 40.0 ± 0.3° and 19.4 ± 1.5°, respectively. In the cTHA cohort, the mean inclination angle in the preoperative plan was 43.1 ± 5.1°, and the anteversion was 17.6 ± 6.4°. The average anteversion in the preoperative plans did not differ substantially between the rTHA and cTHA cohorts. However, the average inclination was significantly different between the two cohorts (*p* < 0.001).

**Table 2:** Average anteversion and inclination angles of each group.

Variables	rTHA	cTHA	*t* (*p*-value)
	Mean ± SD	Mean ± SD	
**Preoperative period**			
Anteversion	19.4 ± 1.5°	17.6 ± 6.4°	1.8 (0.08)
Inclination	40.0 ± 0.3°	43.1 ± 5.1°	−4.0 (<0.001)
**Robot-assisted intraoperative period**			
Anteversion	19.9 ± 2.8°		
Inclination	40.2 ± 2.7°		
**Postoperative period**			
Sagittal version	24.8 ± 6.4°	27.5 ± 11.9°	−1.34 (0.17)
Anteversion	28.7 ± 7.0°	31.1 ± 8.3°	−1.69 (0.10)
Inclination	40.7 ± 4.0°	41.3 ± 7.1°	−0.52 (0.61)
*p*-value Preop vs. post-op measure (inclination)	0.07	0.12	
*p*-value Preop vs. intra-op measure (inclination)	0.54		
*p*-value Preop vs. post-op measure (anteversion)	<0.001	<0.001	
*p*-value Preop vs. intra-op measure (anteversion)	0.43		
**Absolute difference**			
Postoperative CT – preoperative anteversion	9.3 ± 7.1°	13.5 ± 11.7°	−2.22 (0.03)
Postoperative CT – preoperative inclination	0.7 ± 4.0°	−1.8 ± 7.4°	2.11 (0.04)

SD = Standard deviation; rTHA = robotic-assisted total hip arthroplasty; cTHA = conventional total hip arthroplasty; CT = computerised tomography scan of the hip; Preop = preoperative post-op = postoperative; intra-op =intra-operative measurement.

In the rTHA cohort, the mean inclination did not show any significant difference between the preoperative plans and intraoperative records (*p* = 0.54) as well as between postoperative measurements and the preoperative plans (*p* = 0.07). Also, although the mean anteversion did not reveal any significant difference between intraoperative measurements and the preoperative plans (*p* = 0.43); there was a remarkable change in the anteversion between the preoperative plans and postoperative measurements (*p* < 0.001) as shown in Table 2.

In the cTHA cohort, the mean inclination did not show any significant change between the preoperative plans and postoperative assessments (*p* = 0.12); however, there was a remarkable change in the anteversion between postoperative measurements and the preoperative plans (*p* < 0.001) as shown in Table 2.

Additionally, we compared absolute variations in the mean angles between postoperative measurements and preoperative plans of the rTHA and cTHA cohorts and found significant variations in the anteversion (*p* = 0.03) and inclination (*p* = 0.04). This is shown in Table 2.

### Location of acetabular cups of the two cohorts

**Figure 2:**
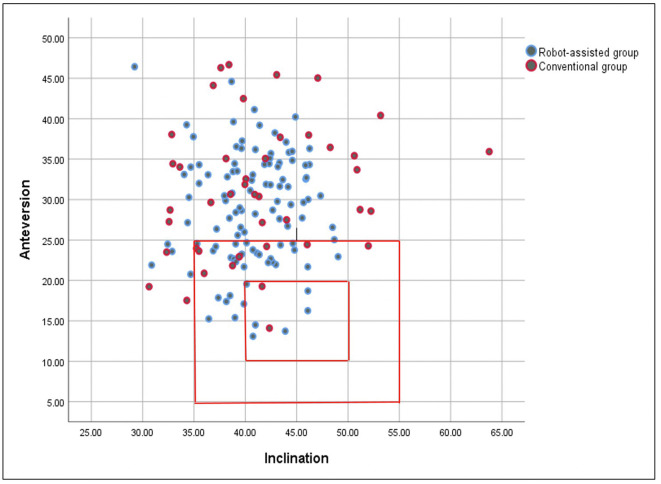
Scatterplot showing the inclination and anteversion angle of each hip with the outer and inner red boxes indicating ±10° and ±5°, respectively of the ideal position.

Figure 2 is a scatterplot showing the inclination and anteversion of each hip with the outer and the inner red boxes indicating ±10° and ±5°, respectively of the ideal position. Overall, Figure 2 showed that in the rTHA cohort, 64.8% of the acetabular cups were located outside the Lewinnek’s safe zone, and 68.5% were located outside the Callanan’s safe zone (Table 3). Also, in the cTHA cohort, 72.1% and 74.4% of the cups were outside the Lewinnek’s and Callanan’s safe zone, respectively. The difference between the cohorts in terms of the proportion of the cups outside the Lewinnek’s (*p* = 0.39) and Callanan’s safe zones (*p* = 0.47) were not significantly different (Table 3).

**Table 3:** A comparison of the number and proportion of acetabular cups placed outside the two referenced safe zones among robot-assisted and conventional THA cohorts.

Variables	Outside Callanan	Outside Lewinnek
	Zone *n* (%)	Zone *n* (%)
**Robot-assisted THA (*N* = 108)**		
Pre-op plan	0 (0)	0 (0)
Intra-op robotic-arm measurement	9 (8.3)	5 (4.6)
Post-op CT-scan measurement	74 (68.5)	70 (64.8)
**Conventional THA (*N* = 43)**		
Pre-op plan	18 (41.8)	9 (20.9)
Post-op CT-scan measurement	32 (74.4)	31 (72.1)

THA = total hip arthroplasty; pre-op = pre-operative, intra-op = intra-operative, post-op = post-operative.

**Figure 3:**
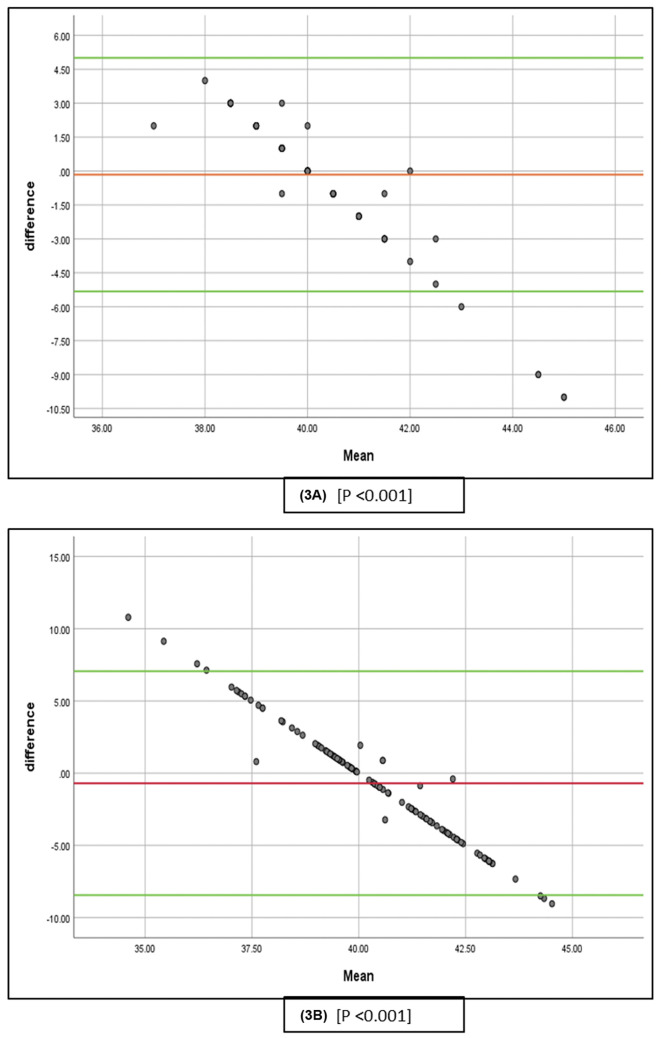
(A) The modified Bland-Altman figure, which includes the 95% prediction interval of the mean, shows the discrepancy between the intraoperatively recorded cup inclination and the preoperative plan. (B) The adjusted Bland-Altman plot shows the 95% confidence interval for the mean and the discrepancy between the postoperatively measured cup inclination and the preoperative plan.

**Figure 4:**
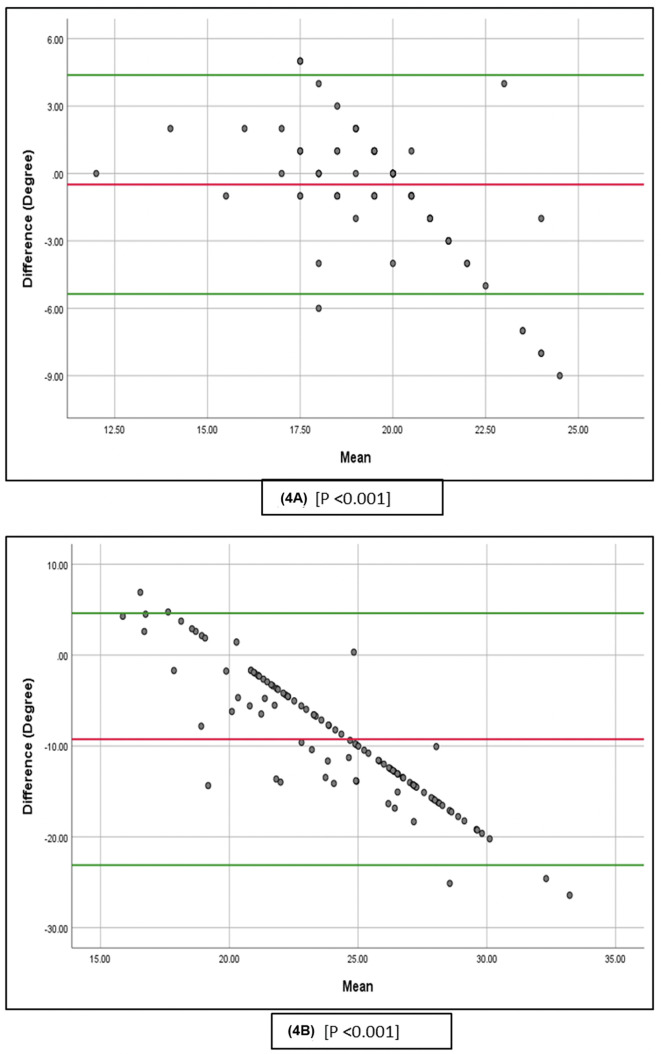
(A) The 95% prediction interval of the mean and the modified Bland-Altman plot of the discrepancy between intraoperatively recorded anteversion of the cup and the preoperative plan. (B) The adjusted Bland-Altman plot shows the 95% confidence interval for the mean and the discrepancy between the postoperatively measured anteversion of the cup and the preoperative plan.

Figures 3 and 4 show the 95% predictive intervals of the cup angles. The 95% predictive intervals utilizing the intraoperative robotic-arm data and the preoperative plan were −5.32 to 5.01° for inclination (Figure 3A) and −5.36 to 4.38° for anteversion (Figure 4A). The predictive intervals for the radiographic measurements (Figures 3B and 4B) were broader: −8.45 to 7.053° for inclination and −23.04 to 4.54° for anteversion. Overall, the predictive interval indicates evidence of proportional bias between the preoperative and intraoperative robotic-assisted records (*p* < 0.001) and also between the postoperative radiographic measurements and preoperative plan (*p* < 0.001) for both the inclination and anteversion of the cup (Figures 3 and 4).

## Discussion

Successfully adopting new technology for THA requires validation to demonstrate its ability to improve patient outcomes. The use of robotic-assisted technology for acetabular cup preparation and implantation with the help of computer-aided tools (such as the MAKO system) is an emerging approach to THA. For the system to improve surgical outcomes, it must be simple to use and provide better accuracy and precision compared to the conventional approach to THA. The key questions investigated by the study were to assess the precision and accuracy of acetabular cup anteversion and inclination intraoperatively as compared to the preoperative planned positions, and as validated by postoperative CT scans. Second, we compared the accuracy of rTHA with a cohort of cTHA patients operated by the same surgeon and analyzed the outliers. There was improved precision for inclination using the robotic system but no improvement was recorded for anteversion postoperatively. Furthermore, when we compared the rTHA with the cTHA cohorts, there were significant differences in the preoperative inclination, but there were no significant differences in the preoperative anteversion and postoperative anteversion and inclination. Also, there were no significant differences in the proportion of acetabular cups of the patients placed outside the two referenced safe zones in the rTHA and cTHA cohorts [8, 9].

This study is crucial because it validates a tool that has the potential to enable surgeons to achieve precise and accurate acetabular component implantation. Studies that adopted the cTHA approach have reported that in 40 to 78% of patients, the inclination and/or anteversion cannot be maintained within Lewinnek’s safe zone [9, 19, 20]. This inability to achieve precision of inclination and anteversion of uncemented acetabular cups is the most common reason for dislocation following THA [2, 21]. Several studies have demonstrated that robotic-assisted instrumentation for acetabular component placement can maintain cup anteversion and inclination within Lewinnek’s safe zone [8, 22–25]. Domb et al. found that with the rTHA approach, 50 out of 50 hips (100%) had cup positions for anteversion and inclination maintained within Lewinnek’s safe zone, compared to a success rate of 80% (40 out of 50) with the cTHA approach [13]. Nodzo et al. in their study of 20 patients reported a significant association between the mean postoperatively measured version (23.0 ± 2.4°; *r*^2^ = 0.76; *p* < 0.001) and intraoperative version (23.2 ± 2.3°). However, they also noted that three participants had CT scan measurements that varied remarkably from the intraoperative robotic measurements [22].

In this study, we found that robotic-assisted THA can be useful for intraoperative decision-making and preoperative planning. However, we observed a marked variation in the postoperative version resulting in a majority of the rTHA cases being located outside Lewinnek’s and Callanan’s safe zones. In addition, we found evidence of proportional bias in the measurements. Although many studies have documented differences ranging from 10 to 20% of outliers when comparing preoperative/intraoperative inclination and anteversion of rTHA cases with the postoperative scan [23–25], our findings indicate a marked variation from these studies. There was no statistically significant association in the evaluation of the inclination of the acetabular components position between the postoperative CT scan and the intraoperative records. However, there was a statistically significant difference in the preoperative and postoperative anteversion measurement of the rTHA cohort. The likelihood of a significant change in cup position in the immediate postoperative period is low. While we cannot definitively explain these discrepancies, several factors have been suggested in the literature as potential contributors. One possible factor is the changes in pelvic orientation that can occur after surgery due to flexion contractures, pain, or muscle spasms [14, 26, 27]. Additionally, some studies have reported that pelvic tilt may continue to change over time in the postoperative period [7, 26–28]; and that there can be substantial differences in the pelvic tilt between standing and supine positions [7, 27, 28]. These functional changes in pelvic alignment may have an impact on acetabular component performance, even when the components were initially placed within the “safe zone.” However, given the scope of this study, it is not possible to confirm whether these factors fully account for the observed discrepancies. We suggest that further research is needed to better understand the underlying causes.

The study has some limitations. There was no randomization between rTHA and cTHA cohorts. However, the prospectively developed registry system which allowed automatic recording and storage of the robotic system data ensured the authenticity of data. Second, the patients in the rTHA cohort were significantly younger than those in the cTHA cohort; and the effect of age was not accounted for in the analysis. Third, the findings of this study were based on a single surgeon’s learning curve which may not be generalizable to other surgeons. A study has shown that the learning curve is moderated by the surgeon’s experience, the complexity of the procedure, and an understanding of the robotic system [24]. Further studies involving multiple surgeons are needed to assess the learning curve and proficiency with the robotic system.

## Conclusions

Robot-assisted systems can be useful for intraoperative decision-making and preoperative planning during THA. The results suggest that while robotic-assisted guidance ensures consistent cup inclination, there may be more variability in achieving the planned anteversion, which warrants further investigation into the factors influencing postoperative changes in acetabular orientation. The adoption of rTHA did not substantially lower acetabular cup malpositioning compared to conventional techniques. Future studies on the role of robotic systems in THA should investigate the reason for the discrepancies in the version of the cup recorded during the postoperative period.

## Data Availability

Available upon request from the corresponding author.
